# Dengue and chikungunya seroprevalence among Qatari nationals and immigrants residing in Qatar

**DOI:** 10.1371/journal.pone.0211574

**Published:** 2019-01-31

**Authors:** John M. Humphrey, Enas S. Al-Absi, Munia M. Hamdan, Sara S. Okasha, Diyna M. Al-Trmanini, Hend G. El-Dous, Soha R. Dargham, John Schieffelin, Laith J. Abu-Raddad, Gheyath K. Nasrallah

**Affiliations:** 1 Division of Infectious Diseases, Department of Medicine, Indiana University, Indianapolis, IN, United States of America; 2 Department of Biomedical Science, College of Health Sciences, Qatar University, Doha, Qatar; 3 BioMedical Research Center, Qatar University, Doha, Qatar; 4 Infectious Disease Epidemiology Group, Weill Cornell Medicine‐Qatar, Cornell University, Qatar Foundation—Education City, Doha, Qatar; 5 Section of Infectious Diseases, Department of Pediatrics, Tulane University School of Medicine, New Orleans, LA, United States of America; 6 Department of Healthcare Policy and Research, Weill Cornell Medicine, Cornell University, New York, NY, United States of America; Faculty of Science, Ain Shams University (ASU), EGYPT

## Abstract

The objective of this study is to characterize the seroprevalence of anti-dengue (DENV) and anti-chikungunya (CHIKV) antibodies among blood donors residing in Qatar who are Middle East and North Africa (MENA) nationals and non-nationals. Sera were collected from adult blood donors in Qatar from 2013 to 2016 and tested for anti-DENV and anti-CHIKV IgG using commercial microplate enzyme-linked immunosorbent assays. Age-specific seroprevalence was summarized by region/nationality: Asia (India, Philippines), Middle East (Iran, Jordan, Lebanon, Pakistan, Palestine, Syria, Yemen), North Africa (Egypt, Sudan), Qatar. The adjusted odds of anti-DENV and anti-CHIKV IgG seropositivity was estimated by logistic regression. Among 1,992 serum samples tested, Asian nationals had higher adjusted odds of being seropositive for anti-DENV antibodies compared to nationals of the Middle East (aOR 0.05, 95% CI 0.04–0.07), North Africa (aOR 0.14, 95% CI 0.10–0.20), and Qatar (aOR 0.01, 95% CI 0.01–0.03). Asian nationals also had higher adjusted odds of being seropositive for anti-CHIKV antibodies compared to those from the Middle East (aOR 0.14, 95% CI 0.07–0.27), North Africa (aOR 0.50, 95% CI 0.26–0.96), and Qatar (aOR 0.38, 95% CI 0.15–0.96). The adjusted odds of being anti-DENV seropositive was higher among anti-CHIKV seropositive adults, and vice versa (aOR 1.94, 95% CI 1.09–3.44), suggesting co-circulation of these viruses. DENV and CHIKV exposure is lower in Qatar and MENA nationals compared to Asian nationals suggesting a lower burden of DENV and CHIKV disease in the MENA. Antibodies to both viruses were detected in nationals from most MENA countries, supporting the need to better understand the regional epidemiology of these viruses.

## Introduction

Although dengue (DENV) and chikungunya (CHIKV) viruses rank among the most important causes of arboviral diseases in the world, their epidemiology in the Middle East and North Africa (MENA) is sparsely characterized [1, 2]. Recent outbreaks of DENV and CHIKV have been documented in several MENA countries including Pakistan, Sudan, and Yemen, while in over half of MENA countries, no seroprevalence data has ever been published [1–8]. In Qatar, neither local transmission of DENV or CHIKV, nor the presence of their principal vectors, *Aedes aegypti* and *Aedes albopictus*, has yet been reported to our knowledge. However, the expansion of DENV in neighboring Saudi Arabia and Yemen underscores the importance of understanding the epidemiology of these pathogens and their potential for spread in Qatar and the surrounding region.

**Fig 1:**
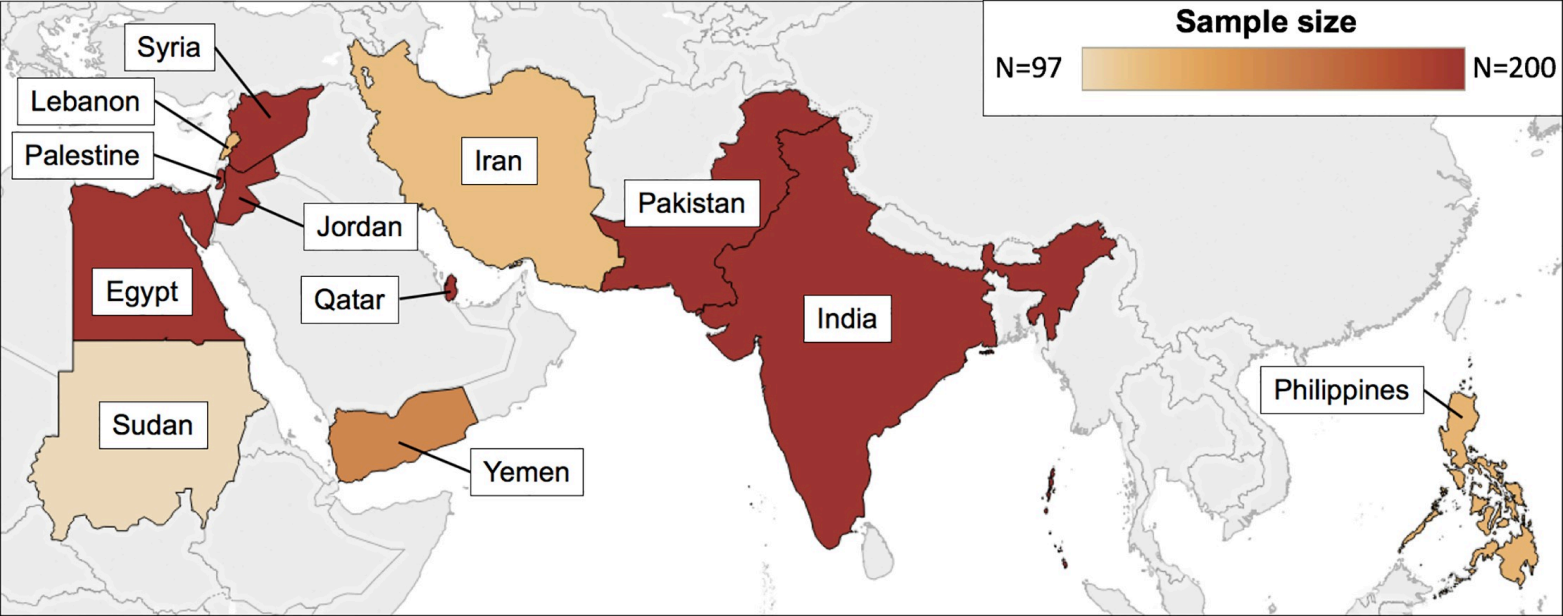
Represented countries (n = 12) among Qatari nationals and immigrants residing in Qatar who were included in the study.

Qatar is situated on the northeastern coast of the Arabian Peninsula, surrounded by the Arabian Gulf and bordering Saudi Arabia to the south (Fig 1). Rainfall is infrequent (<100 mm annually) and average high temperatures often exceed 38°C [9]. Modeling studies have identified low probability of occurrence of *Ae*. *aegypti* in Qatar on account of poor environmental suitability (e.g. high temperature, low precipitation), but potential for *Ae*. *albopictus* occurrence in urban areas of Qatar [10]. Still, Qatar has various characteristics that could influence the potential for local DENV and CHIKV transmission and present a unique opportunity to study the seroepidemiology of these viruses in the MENA. First, 88% of the country’s 2.2 million people are migrants from other countries [11]. Up to 60% of these migrants come from the Indian subcontinent and the Philippines, posing risk of imported infections from some of the most highly DENV and CHIKV endemic countries in the world [12–14]. Second, MENA nationals from Egypt, Pakistan, Syria, Sudan, Jordan, Iran, and Lebanon are estimated to make up approximately 22% of the country’s population [15]. In most of these countries, local DENV or CHIKV transmission, serologic evidence of past infection, or the presence of Ae. *aegypti* or *Ae*. *albopictus* has been documented, yet published epidemiologic data is lacking.

Blood donor serosurveys can be an efficient means of gaining preliminary insight into the epidemiology and potential burden of these viruses in this circumstance. Few blood donor surveys have ever been conducted for DENV and CHIKV in the MENA region, and none have sampled nationals from other countries or compared prevalence to non-MENA nationals [16–22]. The objective of this study is to characterize the seroprevalence of anti-DENV and anti-CHIKV antibodies among blood donors residing in Qatar who are MENA and non-MENA nationals. Such data will address knowledge gaps in our understanding of the seroepidemiology of these pathogens in the MENA region, and their potential risk of emergence in Qatar.

## Methods

### Ethics statement

The research work was approved by the ethics boards and research committees at Qatar University, Hamad Medical Corporation, and Weill Cornell Medicine-Qatar. The requirement for informed consent was waived by these institutions given that samples were already de-identified at the time they were received for this research. All experiments were performed in accordance with relevant guidelines and regulations.

### Study design and participants

This was a retrospective, cross-sectional study using de-identified blood samples collected from volunteer blood donors attending Hamad Medical Corporation in Qatar, the largest healthcare provider in the country, from June 2013 to June 2016. In total, 5,973 blood donors consented to submit blood specimens and basic demographic information (age, nationality, gender) which were analyzed in other studies [23–28]. A subset of this biobank, selected at random, was analyzed for this study. The original sample set included male and female Qataris and expatriates (MENA and non-MENA nationals) who were ≥ 18 years of age and residing in Qatar. In total, there were 5,799 men and 152 women, with gender type missing in 0.4% (n = 22) of participants, which served as the original sampling cohort for selection of the final sample. Given the low number of available samples from women, only samples from men were included in our study.

Sample sizes were calculated for a significance level of α = 0.05. A minimum sample size of 100 was estimated for each country based on a projected overall anti-DENV antibody seroprevalence of 25% with 9% precision and an anti-CHIKV antibody prevalence of 1% with 2% precision [1, 2]. However, up to 200 samples were analyzed per country based on sample availability in order to increase the precision of the estimates. Attrition did not need to be accounted for, as it is minimal, given that a sufficient quantity of blood donor samples was known to be available from our prior studies of the seroprevalence of herpes simplex viruses [27, 28].

### Biological sample collection and laboratory analysis

A total of 20 μL of serum was aliquoted from each participant’s sample that had been stored at -80°C. Sera were tested for the presence of anti-DENV and anti-CHIKV IgG (10 μL serum for each test) using commercial microplate enzyme linked immunosorbent assay (ELISA) kits for anti-DENV (NovaTec Immundiagnostica GmbH, Frankfurt, Germany, DENG0120) and anti-CHIKV (Euroimmun, Lübeck, Germany, EI 293a-9601 G) antibodies [29, 30]. These kits are designed for monospecific determination of IgG antibodies with 3-point quantitative calibration and approved by the United States Food and Drug Administration. As per the manufacturer, the anti-DENV IgG ELISA is based on purified virus particles of serotype 2, allowing for detection of virus serotypes 1–4 based on the structural similarities between them [31]. The sensitivity and specificity was 100% and 97%, respectively, in clinically characterized sera, though cross-reactions with other flaviviruses can occur [20]. The anti-CHIKV ELISA uses a virus-specific structural protein as the antigenic substrate with a sensitivity and specificity of 95% and 88%, respectively [32]. Cross-reactions against o’nyong-nyong virus and Mayaro virus were observed in this latter study [32].

### Data analysis

For each virus, the proportion of positive samples was summarized with 95% confidence intervals (CI) according to nationality and age across seven bands: ≤ 24, 25–29, 30–34, 35–39, 40–44, 45–49, and ≥ 50 years. Age-specific seroprevalence for each virus was also cross-tabulated by region: Asia (India, Philippines), Middle East (Iran, Jordan, Lebanon, Pakistan, Palestine, Syria, Yemen), North Africa (Egypt, Sudan), and Qatar. Regions were defined according to the MENA definitions of the World Health Organization Regional Office for the Eastern Mediterranean (WHO/EMRO), the Joint United Nations Programme on HIV/AIDS (UNAIDS), and the Word Bank, and for consistency with our earlier dengue, chikungunya, and HIV regional publications [1, 2, 33–36]. Associations between nationality, age and seropositivity were summarized with odds ratios (ORs) and 95% CIs. Logistic regression was used to estimate the adjusted effects of age and regional nationality on the odds of seropositivity for anti-DENV and anti-CHIKV IgG separately, as well as the odds of anti-DENV IgG positivity in the setting of anti-CHIKV IgG positivity, and vice versa. Significance level was defined at α = 0.05. Data was analyzed using the Statistical Package for the Social Sciences (SPSS) version 24.

## Results

A total of 1,992 serum samples from male subjects ≥ 18 years of age from 12 countries were tested for anti-DENV and anti-CHIKV IgG antibodies (Fig 1 and Table 1). The number of samples tested per country ranged from 97 (< 100 due to insufficient serum) to 200. The median [IQR] age of subjects was 36 [30–43] years. Anti-DENV IgG was detected in six or more samples from individuals from every country, while anti-CHIKV IgG was detected in at least one sample from individuals from every country except Iran. The country-specific overall seroprevalence for anti-DENV IgG ranged from 3.5% (95% CI 1.6–6.8%) in Qatar to 95.8% (95% CI 91.0–98.4%) in the Philippines. For anti-CHIKV IgG, overall seroprevalence ranged from 0% in Iran to 17.7% (95% CI 11.6–25.2%) in the Philippines.

**Table 1:** Country-specific seroprevalence for anti-DENV IgG and anti-CHIKV IgG among subjects residing in Qatar but from different countries, from June 2013 to June 2016.

Country	Sample size	Prevalence			
		Anti-DENV IgG positive	% (95% CI)	Anti-CHIKV IgG positive	% (95% CI)
Egypt	199	40	20.1 (15.0–26.1)	11	5.5 (3.0–9.4)
India	200	125	62.5 (55.7–70.0)	22	11.0 (7.2–15.9)
Iran	113	6	5.3 (2.3–10.6)	0	0 (0)
Jordan	199	9	4.5 (2.3–8.1)	1	0.5 (0.1–2.3)
Lebanon	116	6	5.2 (2.2–10.4)	1	0.9 (0.1–4.0)
Pakistan	200	40	20.0 (14.9–26.0)	3	1.5 (0.3–4.3)
Palestine	200	17	8.5 (5.2–13.0)	6	3.0 (1.2–6.1)
Philippines	119	114	95.8 (91.0–98.4)	21	17.7 (11.6–25.2)
Qatar	200	7	3.5 (1.6–6.8)	7	3.5 (1.6–6.8)
Sudan	97	47	48.5 (38.7–58.3)	5	5.2 (2.0–10.9)
Syria	200	26	13.0 (8.9–18.2)	1	0.5 (0.1–2.3)
Yemen	149	36	24.2 (17.8–31.5)	4	2.7 (0.9–6.3)

The overall and age-specific seroprevalence for each virus was also estimated for nationals residing in Qatar but from Asia, Middle East, North Africa, and Qatar (Tables 2 and 3). Age-specific seroprevalence values were significantly different across regions for both viruses in all age groups (*P <* 0.05 for all), with the exception of anti-CHIKV IgG seroprevalence for adults ≥ 50 years (*P =* 0.20). Overall, the highest seroprevalence estimates for anti-DENV IgG occurred among Asian nationals (74.8% seropositive, 95% CI 69.7–79.5%) and the lowest among Qatari nationals (3.5% seropositive, 95% CI 1.4–7.1%). For anti-CHIKV IgG, the highest seroprevalence also occurred among Asian nationals (13.5% seropositive, 95% CI 10.0–17.8%), and the lowest among Middle East nationals (1.4% seropositive, 95% CI 0.8–2.2%).

**Table 2:** Estimates of age-specific anti-DENV IgG seroprevalence among blood donors currently residing in Qatar but from Asia (India, Philippines), Middle East (Iran, Jordan, Lebanon, Pakistan, Palestine, Syria, Yemen), North Africa (Egypt, Sudan), and Qatar, from June 2013 to June 2016.

Age group	Asia^a^		Middle East^b^		North Africa		Qatar		P value^c^
Years	N+ / Total	% (95% CI)	N+ / Total	% (95% CI)	N+ / Total	% (95% CI)	N+ / Total	% (95% CI)	
≤ 24	7 / 15	46.7 (23.9–70.6)	9 / 111	8.1 (4.1–14.3)	2 / 19	10.5 (2.3–29.7)	0 / 16	0 (0–0)	<0.001
25–29	42 / 56	75.0 (62.6–84.9)	12 / 167	7.2 (4.0–11.9)	22 / 61	36.1 (24.9–48.5)	0 / 33	0 (0–0)	<0.001
30–34	65 / 85	76.5 (66.7–85.0)	31 / 238	13.0 (9.2–17.7)	17 / 64	26.6 (17.0–38.3)	2 / 35	5.7 (1.2–17.1)	<0.001
35–39	47 / 61	77.1 (65.4–86.2)	29 / 231	12.6 (8.8–17.3)	20 / 66	30.3 (20.2–42.1)	1 / 38	2.6 (0.3–11.7)	<0.001
40–44	32 / 43	74.4 (60.1–85.6)	23 / 198	11.6 (7.7–16.6)	13 / 36	36.1 (22.0–52.4)	2 / 35	5.7 (1.2–17.1)	<0.001
45–49	32 / 41	78.1 (63.8–88.6)	13 / 111	11.7 (6.7–18.7)	4 / 20	20.0 (7.2–40.8)	2 / 20	10.0 (2.1–28.4)	<0.001
≥ 50	13 / 17	76.5 (53.3–91.5)	23 / 119	19.3 (13.0–27.1)	9 / 30	30 (16.0–47.7)	0 / 23	0 (0–0)	<0.001
**Total**	238 / 318	74.8 (69.7–79.5)	140 / 1175	11.9 (10.1–13.9)	87 / 296	29.4 (24.3–34.9)	7 / 200	3.5 (1.4–7.1)	<0.001

“N+” indicates number of positive samples.

^a^ Age information was missing for one individual.

^b^ Age information was missing for two individuals.

^c^ P value for differences between regions.

**Table 3:** Estimates of age-specific anti-CHIKV IgG seroprevalence among blood donors currently residing in Qatar but from Asia (India, Philippines), Middle East (Iran, Jordan, Lebanon, Pakistan, Palestine, Syria, Yemen), North Africa (Egypt, Sudan), and Qatar, 2013–2016.

Age group	Asia^a^		Middle East^b^		North Africa		Qatar		P value^c^
Years	N+ / Total	% (95% CI)	N+ / Total	% (95% CI)	N+ / Total	% (95% CI)	N+ / Total	% (95% CI)	
≤ 24	2 / 15	13.3 (2.9–36.3)	2 / 111	1.8 (0.4–5.7)	0 / 19	0 (0–0)	0 / 16	0 (0–0)	0.039
25–29	7 / 56	12.5 (5.8–23.0)	4 / 167	2.4 (0.8–5.6)	3 / 61	4.9 (1.4–12.6)	1 / 33	3.0 (0.3–13.3)	0.026
30–34	10 / 85	11.8 (6.21–19.9)	3 / 238	1.3 (0.4–3.3)	4 / 64	6.3 (2.2–14.2)	3 / 35	8.6 (2.5–21.1)	<0.001
35–39	5 / 61	8.2 (3.2–17.0)	1 / 231	0.4 (0.1–2.0)	4 / 66	6.1 (2.1–13.8)	0 / 38	0 (0–0)	0.001
40–44	9 / 43	20.9 (10.9–34.7)	3 / 198	1.5 (0.4–4.0)	1 / 36	2.8 (0.3–12.3)	1 / 35	2.9 (0.3–12.6)	<0.001
45–49	8 / 41	19.5 (9.7–33.5)	0 / 111	0 (0–0)	3 / 20	15.0 (4.4–34.9)	1 / 20	5 (0.5–21.1)	<0.001
≥ 50	2 / 17	11.8 (2.5–32.7)	3 / 119	2.5 (0.7–6.6)	1 / 30	3.3 (0.4–14.5)	1 / 23	4.3 (0.5–18.6)	0.203
**Total**	43 / 318	13.5 (10.0–17.8)	16 / 1175	1.4 (0.8–2.2)	16 / 296	5.4 (3.1–8.6)	7 / 200	3.5 (1.4–7.1)	<0.001

“N+” indicates number of positive samples.

^a^ Age information was missing for one individual.

^b^ Age information was missing for two individuals.

^c^ P value for differences between regions.

Regional nationality was significantly associated with the odds of being anti-DENV and anti-CHIKV IgG seropositive in the unadjusted and adjusted models (*P* < 0.05 for all; Table 4).

**Table 4:** Unadjusted and adjusted odds ratios for anti-DENV IgG and anti-CHIKV IgG seropositivity among blood donors residing in Qatar but from different regions.

Characteristic	Unadjusted Odds Ratio				Adjusted Odds Ratio^a^			
	Anti-DENV IgG UOR (95% CI)	P value	Anti-CHIKV IgG UOR (95% CI)	P value	Anti-DENV IgG AOR (95% CI)	P value	Anti-CHIKV IgG AOR (95% CI)	P value
Region								
Asia	Ref		Ref		Ref		Ref	
Middle East	0.05 (0.03–0.06)	<0.001	0.09 (0.05–0.16)	<0.001	0.05 (0.04–0.07)	<0.001	0.14 (0.07–0.27)	<0.001
North Africa	0.14 (0.10–0.20)	<0.001	0.37 (0.20–0.67)	0.001	0.14 (0.10–0.20)	<0.001	0.50 (0.26–0.96)	0.038
Qatar	0.01 (0.01–0.03)	<0.001	0.23 (0.10–0.53)	<0.001	0.01 (0.01–0.03)	<0.001	0.38 (0.15–0.96)	0.041
Age group (years)								
≤ 24	Ref		Ref		Ref		Ref	
25–29	2.51 (1.44–4.36)	0.001	1.95 (0.64–5.97)	0.243	1.20 (1.05–3.79)	0.035	1.23 (0.39–3.89)	0.721
30–34	2.98 (1.74–5.08)	<0.001	1.95 (0.66–5.80)	0.229	2.38 (1.28–4.41)	0.006	1.21 (0.40–3.70)	0.740
35–39	2.58 (1.50–4.43)	0.001	1.02 (0.31–3.29)	0.978	2.42 (1.30–4.51)	0.006	0.69 (0.21–2.29)	0.541
40–44	2.30 (1.32–4.01)	0.003	1.84 (0.60–5.70)	0.288	2.40 (1.25–4.56)	0.008	1.42 (0.45–4.52)	0.551
45–49	2.87 (1.60–5.16)	<0.001	2.62 (0.83–8.28)	0.102	2.26 (1.13–4.50)	0.021	1.65 (0.51–5.42)	0.406
≥ 50	2.48 (1.37–4.50)	0.003	1.51 (0.43–5.25)	0.517	3.18 (1.61–6.28)	0.001	1.28 (0.36–4.59)	0.704
Anti-DENV IgG positive	—	—	4.68 (2.98–7.34)	<0.001	—	—	1.94 (1.09–3.44)	0.024
Anti-CHIKV IgG positive	4.68 (2.98–7.34)	<0.001	—	—	1.94 (1.09–3.44)	0.024	—	—

^a^ Adjusted for nationality, age, and other arbovirus seropositivity (i.e. anti-DENV seropositivity status as predictor of anti-CHIKV seropositivity, and vice versa). For this analysis, n = 1,989 given missing age information for three individuals.

Asian nationals had higher adjusted odds of being seropositive for anti-DENV antibodies compared to nationals of the Middle East (aOR 0.05, 95% CI 0.04–0.07), North Africa (aOR 0.14, 95% CI 0.10–0.20), and Qatar (aOR 0.01, 95% CI 0.01–0.03). Asian nationals also had higher adjusted odds of being seropositive for anti-CHIKV antibodies compared to those from the Middle East (aOR 0.14, 95% CI 0.07–0.27), North Africa (aOR 0.50, 95% CI 0.26–0.96), and Qatar (aOR 0.38, 95% CI 0.15–0.96). Increasing age was also significantly associated with anti-DENV IgG seroprevalence, with individuals ≥ 50 years of age having 3.18 times (95% CI 1.61–6.28) the adjusted odds of being anti-DENV IgG seropositive compared to individuals ≤ 24 years of age (Table 4). However, age was not significantly associated with the adjusted odds of seropositivity for anti-CHIKV IgG. Finally, the adjusted odds of being anti-DENV IgG seropositive was significantly higher among anti-CHIKV IgG seropositive adults, and vice versa (aOR 1.94, 95% CI 1.09–3.44).

## Discussion

In our study, anti-DENV and anti-CHIKV IgG prevalences were significantly lower among blood donors residing in Qatar who were MENA nationals compared to Asian nationals, though antibodies to both viruses were detected from donors in all regions. This finding underscores the need to better understand the distribution and epidemiology of these pathogens in the MENA region, but it suggests that the overall burden of DENV and CHIKV disease is higher in India and the Philippines than in the MENA countries represented in our study. The results also suggest regional variability within the MENA region.

Our study is the first, to our knowledge, to estimate the seroprevalence of anti-DENV and anti-CHIKV IgG among nationals of Qatar and Syria [1, 2]. We cannot be certain that individuals from these countries developed antibodies as a result of DENV and CHIKV exposure in their home countries or Qatar rather than through traveling or residing in other endemic countries, or that these antibodies definitively represent prior exposure and not cross-reactions to other pathogens. Nevertheless, these detections suggest the possibility of low, but potentially unrecognized, transmission of DENV and CHIKV in these MENA countries.

Consistent with many other studies, older age was significantly associated with increased odds of DENV exposure in our study [37–40]. Immunity for the infecting DENV serotype (i.e. detection of serum neutralizing antibodies) is considered to be lifelong, though homotypic reinfections (i.e. reinfection by the same serotype) can occur [41, 42]. Hence, the increased odds of DENV exposure with age is in part a function of longer exposure time. Age was not associated with CHIKV exposure in our study, though this association has been observed for CHIKV [37]. Detection of this association may have been limited by the small proportion of CHIKV seropositive samples in our study and that many (41%) participants were 30–39 years of age (generally reflective of the migrant demographic in Qatar) with variable sample sizes in each age group. As a secondary analysis, we grouped countries by overall seroprevalence levels: high (>30% seroprevalence: India, Philippines, Sudan), medium (15–30% seroprevalence: Egypt, Pakistan, Yemen) and low (<15% seroprevalence: Iran, Jordan, Lebanon, Palestine, Qatar, Syria). These seroprevalence values represent practical cutoffs based on the country-level seroprevalences in our study, as there is currently no consensus to classify population seroprevalence as high or low for either virus to our knowledge. However, this did not result in meaningful changes in any of the aforementioned associations compared to the primary analysis. Finally, the adjusted odds of anti-DENV IgG seropositivity was nearly twice as high among anti-CHIKV IgG seropositive individuals compared to seronegative individuals, which is consistent with the co-circulation of these viruses as a result of their shared vectors *Ae*. *aegypti* and *Ae*. *albopictus*, and suggests the common source exposure to subjects for both pathogens.

### Dengue in the MENA region

Anti-DENV IgG antibodies were identified among nationals of all countries in our study, with the highest prevalence among Asian nationals (India 62.5% and Philippines 95.8%). Among MENA nationals, those from Sudan (48.5%), Yemen (24.2%), and Pakistan (20.0%) had the highest seroprevalence. This is consistent with the epidemiologic literature, as available studies suggest DENV is widely endemic in Sudan with seroprevalence of 9–49% among general populations, and multiple outbreaks have occurred along the Red Sea coast since the 1980s [5, 43–47]. The risk of DENV exposure may be similarly high in Yemen, in which recent outbreaks have resulted in 19–87% seroprevalence among general populations [8, 48–51]. In Pakistan, serologic and outbreak data suggest DENV is distributed across the country [52–56].

Our present study also identified 5–13% seroprevalence among nationals from Lebanon, Palestine, and Syria, in which no reports of DENV have been published in decades [57, 58]. However, *Ae*. *albopictus* and/or *Ae*. *aegypti* have been recently reported in these countries, and there are historic reports of DENV transmission in Lebanon, raising the potential for unrecognized transmission or future emergence in these countries [57, 59, 60]. Additionally, our study identified 20.1% seroprevalence among Egyptian nationals. *Ae*. *aegypti* is known to be endemic in Egypt and an outbreak was reported in 2015 along the Red Sea Coast, although there is currently a paucity of literature describing the epidemiology of DENV in the country [61–63]. In Jordan, anti-DENV IgG was recently detected in 24% of 892 healthy individuals distributed across the country, representing the first report of anti-DENV seroprevalence in Jordan [64]. As with our data, these data are not direct evidence for local DENV transmission in Jordan, but suggest its possibility. Finally, our study identified 5.3% seroprevalence among Iran nationals, despite no published reports to our knowledge of DENV transmission or *Ae*. *aegypti* occurrence in Iran. However, prior studies have shown 3–7% seroprevalence near the Pakistan border of the country [17, 65, 66]. Vector surveillance is critical in these countries, both to evaluate the risk of DENV transmission and to implement preventive vector control strategies and active case surveillance as indicated.

### Chikungunya in the MENA region

Seroprevalence estimates for anti-CHIKV IgG were significantly lower than anti-DENV IgG estimates in all countries in our study, which is consistent with the current serologic evidence in the MENA region and suggests a lower overall burden of CHIKV disease compared to DENV [2]. Yet although the CHIKV seropositivity was proportionally smaller, the country-level anti-CHIKV IgG proportions were similarly distributed to those of anti-DENV IgG in that those nationals with the highest CHIKV exposure (Philippines, India, Sudan) also had the highest DENV exposure. This supports an overlapping distribution of DENV and CHIKV among the countries represented in our study, on account of the viruses’ shared mosquito vectors, *Ae*. *aegypti* and *Ae*. *albopictus* [13].

Our study identified 0.5–3.0% anti-CHIKV IgG seroprevalence in Jordan, Lebanon, Palestine and Syria. There have been no published reports of CHIKV transmission in these countries to our knowledge. Given the additional lack of confirmed DENV transmission in these countries, this raises the probability that these results are either false-positive and/or represent cross-reactions with other pathogens, such as alphaviruses related to CHIKV (o’nyong-nyong virus, Semliki Forest virus, and Sindbis virus), which are also known to be endemic in the MENA region [16, 18, 65, 67–69]. CHIKV is known to circulate in Pakistan, Sudan, and Yemen, and several anti-CHIKV serologic surveys and outbreaks have been published [7, 8, 50, 67, 70, 71]. Anti-CHIKV antibodies have also been reported from Egypt in the 1970s and 80s, and the country has experienced recent DENV outbreaks and is known to harbor *Ae*. *aegypti* [2, 72]. However, to our knowledge, no CHIKV outbreaks have yet been reported in Egypt.

### Anti-DENV and anti-CHIKV IgG seroprevalence in Qatar

A seroprevalence of 3.5% for both anti-DENV and anti-CHIKV IgG was detected among Qatar nationals in our study despite that neither *Ae*. *aegypti*, *Ae*. *albopictus*, nor autochthonous transmission of either virus, have ever been reported in Qatar to our knowledge. This may represent travel-acquired infections and/or cross-reactions with related viruses. This low seroprevalence does not substantiate the need for routine DENV vaccination in Qatar, particularly given the recommendation that DENV seroprevalence be ≥ 70% in the age group targeted for vaccination to maximize cost-effectiveness and public health impact [73]. Moreover, the risk of transfusion-transmitted DENV or CHIKV through contaminated blood products is likely low given that the presence of IgG likely represents past exposure after the viremic phase of infection has resolved, and that our samples were not collected in a known outbreak setting [74]. Nevertheless, ongoing surveillance for *Ae*. *aegypti* and *Ae*. *albopictus* is warranted in the country, given that vector control remains the primary strategy for outbreak prevention and control, and in light of the recent outbreaks that have occurred in neighboring Saudi Arabia and Yemen [4, 8, 49, 75]. The low seropevalence among Qatari nationals indicates a lack of herd immunity and susceptibility to DENV and CHIKV outbreaks.

Our study has several strengths but also important limitations. Strengths of our study include its large sample size and diverse population. Not only were we able to estimate anti-DENV and anti-CHIKV IgG seroprevalence among adults from MENA countries in which major epidemiologic knowledge gaps exist, but we were also able to compare estimates against Asian nationals from hyper-endemic countries known to carry a considerable burden of DENV and CHIKV disease. A major limitation of our study is our reliance on IgG ELISA and lack of confirmatory viral neutralization testing, the gold standard for serodiagnosis of arboviruses, which was not performed due to resource limitations. Viral neutralization was performed in only 5% of anti-DENV and 17% of anti-CHIKV serologic studies in the MENA region in our systematic reviews, with cross reactions to antigenically similar viruses identified in multiple studies resulting in lower attributable prevalence [1, 2]. Documented anti-DENV antibody cross-reaction with West Nile virus, yellow fever virus (natural and vaccine), Zika virus (e.g. in India and Philippines), or other flaviviruses may account for some of the observed seropositivity in our study, while for anti-CHIKV antibodies, cross-reactions to o’nyong-nyong virus, Semliki Forest virus, and Sindbis virus may have occurred. The lack of travel history limits conclusions that can be made regarding other countries, where DENV and CHIKV exposure may have occurred. The lack of IgM or PCR testing limits inference concerning the timing of infections or occurrences of co-infections. Finally, our limited sample of adult male migrants residing in Qatar may not be representative of the general populations in their respective countries (including females), nor adequately matched to one another for the region-level analyses, although the vast majority of these migrants arrived in Qatar in recent years [27, 28]. Given these limitations, caution must be maintained in extrapolating the serologic results in our study to the broader country or region levels [15].

## Conclusion

Exposure to DENV and CHIKV is low among Qatar and other MENA nationals compared to Asian nationals, suggesting a lower burden of DENV and CHIKV disease in the MENA countries. Antibodies to both viruses were detected in nationals from all MENA countries except Iran, supporting the need for further research to understand the epidemiology of DENV and CHIKV, and the co-circulating viruses that cause serologic cross-reactions, in the MENA. The findings in our study do not support the need for travellers to Qatar to take measures to prevent DENV and CHIKV infections while in Qatar. Surveillance for *Ae*. *aegypti and Ae*. *albopictus* should be implemented in countries in which autochthonous transmission of DENV or CHIKV has not yet been reported.

## Supporting information

S1 Table Raw dataset containing anti-DENV and anti-CHIKV IgG results. (XLSX)
